# Clinical Evaluation of Self-Collected Saliva by Quantitative Reverse Transcription-PCR (RT-qPCR), Direct RT-qPCR, Reverse Transcription–Loop-Mediated Isothermal Amplification, and a Rapid Antigen Test To Diagnose COVID-19

**DOI:** 10.1128/JCM.01438-20

**Published:** 2020-08-24

**Authors:** Mayu Nagura-Ikeda, Kazuo Imai, Sakiko Tabata, Kazuyasu Miyoshi, Nami Murahara, Tsukasa Mizuno, Midori Horiuchi, Kento Kato, Yoshitaka Imoto, Maki Iwata, Satoshi Mimura, Toshimitsu Ito, Kaku Tamura, Yasuyuki Kato

**Affiliations:** a Self-Defense Forces Central Hospital, Tokyo, Japan; b Department of Infectious Disease and Infection Control, Saitama Medical University, Saitama, Japan; c Department of Infectious Diseases, International University of Health and Welfare Narita Hospital, Chiba, Japan; UNC School of Medicine

**Keywords:** SARS-CoV-2, saliva, RT-qPCR, RT-LAMP, antigen test

## Abstract

The clinical performances of six molecular diagnostic tests and a rapid antigen test for severe acute respiratory syndrome coronavirus 2 (SARS-CoV-2) were clinically evaluated for the diagnosis of coronavirus disease 2019 (COVID-19) in self-collected saliva. Saliva samples from 103 patients with laboratory-confirmed COVID-19 (15 asymptomatic and 88 symptomatic) were collected on the day of hospital admission. SARS-CoV-2 RNA in saliva was detected using a quantitative reverse transcription-PCR (RT-qPCR) laboratory-developed test (LDT), a cobas SARS-CoV-2 high-throughput system, three direct RT-qPCR kits, and reverse transcription–loop-mediated isothermal amplification (RT-LAMP). The viral antigen was detected by a rapid antigen immunochromatographic assay. Of the 103 samples, viral RNA was detected in 50.5 to 81.6% of the specimens by molecular diagnostic tests, and an antigen was detected in 11.7% of the specimens by the rapid antigen test. Viral RNA was detected at significantly higher percentages (65.6 to 93.4%) in specimens collected within 9 days of symptom onset than in specimens collected after at least 10 days of symptoms (22.2 to 66.7%) and in specimens collected from asymptomatic patients (40.0 to 66.7%). Self-collected saliva is an alternative specimen option for diagnosing COVID-19. The RT-qPCR LDT, a cobas SARS-CoV-2 high-throughput system, direct RT-qPCR kits (except for one commercial kit), and RT-LAMP showed sufficient sensitivities in clinical use to be selectively used in clinical settings and facilities. The rapid antigen test alone is not recommended for an initial COVID-19 diagnosis because of its low sensitivity.

## Abstract (precis)

The clinical performances of six molecular diagnostic tests and a rapid antigen test for severe acute respiratory syndrome coronavirus 2 (SARS-CoV-2) were clinically evaluated for the diagnosis of coronavirus disease 2019 (COVID-19) in self-collected saliva. Saliva samples from 103 patients with laboratory-confirmed COVID-19 (15 asymptomatic and 88 symptomatic) were collected on the day of hospital admission. SARS-CoV-2 RNA in saliva was detected using a quantitative reverse transcription-PCR (RT-qPCR) laboratory-developed test (LDT), a cobas SARS-CoV-2 high-throughput system, three direct RT-qPCR kits, and reverse transcription–loop-mediated isothermal amplification (RT-LAMP).

## INTRODUCTION

Coronavirus (CoV) disease 2019 (COVID-19), which is caused by severe acute respiratory syndrome CoV 2 (SARS-CoV-2), was first reported in 2019 in Wuhan, China, and the World Health Organization subsequently declared it a pandemic (1; https://www.who.int/emergencies/diseases/novel-coronavirus-2019). The large number of patients with COVID-19 during outbreaks is overwhelming the capacity of national health care systems; therefore, the quick and accurate identification of patients requiring supportive therapies and isolation is important for the management of COVID-19.

The quantitative reverse transcription-PCR (RT-qPCR) assay for SARS-CoV-2 using upper and lower respiratory tract specimens (nasopharyngeal swab, throat swab, and sputum) is the gold standard for diagnosing COVID-19 (2). Laboratory-developed tests (LDT), including RT-qPCR, a high-throughput RT-qPCR system (fully automated from RNA extraction to reporting of results without the need for highly skilled laboratory technicians), and direct rapid RNA extraction-free RT-qPCR kits (using a modified RT-qPCR master mix), have been widely used worldwide (3). Other molecular diagnostic methods, such as reverse transcription–loop-mediated isothermal amplification (RT-LAMP), have also been reported as useful for diagnosing COVID-19 in settings of point-of care testing (4, 5). Recently, a SARS-CoV-2 rapid antigen test (RAT) (Espline SARS-CoV-2; Fuji Rebio Inc., Tokyo, Japan), which combines immunochromatography with an enzyme immunoassay to detect the viral nucleocapsid (N) protein, has been approved by the Japanese government (6). The RAT is beginning to be used for diagnosing COVID-19 in clinical settings because it does not require special equipment, it does not have a time-consuming protocol, and highly skilled laboratory technicians are not essential. Although these diagnostic tests are useful in the identification of patients with COVID-19, the process of collecting upper and lower respiratory tract specimens increases the risk of exposure to viral droplets, and there is a patient burden (7). Therefore, an alternative specimen, which can be self-collected, to diagnose COVID-19 is desirable for the clinical management of COVID-19 during this pandemic era (7).

Previously reported sensitivities of RT-qPCR assays were lower for upper respiratory specimens (32 to 61% for pharyngeal swabs and 63 to 73% for nasopharyngeal swabs) than they were for lower respiratory specimens (72 to 93% for sputum and 93 to 100% for bronchoalveolar lavage fluid) (8–10). Recently, several reports highlighted the clinical usefulness of RT-qPCR analysis of saliva specimens (11–17). Saliva specimens can be easily collected by the patients themselves by spitting into a collection tube; thus, using saliva specimens can reduce the burden on a patient, reduce the risk of exposure to viral droplets for medical workers, and reduce the time (1.38-fold shorter) and cost (2.09-fold lower) of the testing procedure compared to those for nasopharyngeal swabs (18). However, the clinical usefulness of saliva specimens for diagnosing COVID-19 remains controversial because the reported diagnostic sensitivity varies widely between 69.2 and 100%, and it has yet to be thoroughly evaluated due to small sample sizes and a lack of detailed clinical information (11–17, 19).

Here, we describe the clinical performance of various molecular diagnostic methods, including the RT-qPCR LDT, the cobas SARS-CoV-2 high-throughput system, 3 direct RT-qPCR kits, and RT-LAMP, and a commercial SARS-CoV-2 RAT used on self-collected saliva specimens in diagnosing COVID-19.

## MATERIALS AND METHODS

### Patients and sample collection.

Patients with COVID-19 were enrolled in this study after being referred to the Self-Defense Forces Central Hospital in Japan for isolation and treatment under the Infectious Disease Control Law in effect from 11 February to 13 May 2020. All patients were examined for SARS-CoV-2 by RT-qPCR using pharyngeal and nasopharyngeal swabs collected at public health institutes or hospitals in accordance with the nationally recommended method in Japan (20). Asymptomatic patients were tested by RT-qPCR because of mass screening due to an outbreak or family cluster. Patient information was retrospectively collected from the hospital electronic medical records. On the day of admission, a sterile tube was provided for the patients, and they were requested to self-collect saliva specimens (∼500 μl) by spitting into the tube. The saliva specimens were collected without restriction on timing or food intake. All samples were stored at −80°C until sample preparation. All sample preparation and sample analysis were conducted by SRL, Inc. (Tokyo, Japan).

### Sample preparation.

Saliva specimens were diluted with 1- to 5-fold (average, 4-fold) phosphate-buffered saline for an endpoint volume of 2,000 μl. This reduced the viscosity of the sample, allowing for easier pipetting. The suspension was centrifuged at 2,000 × *g* for 5 min at 4°C, and the supernatant was used in the RT-qPCR LDT, cobas SARS-CoV-2 test, RT-LAMP, and RAT on the same day. Residual supernatant was frozen at – 80°C for 4 days until used in the three direct RT-qPCR kit assays.

### Detection of viral RNA by the RT-qPCR LDT using the standard protocol.

The RT-qPCR LDT was performed according to the National Institute of Infectious Diseases (NIID) protocol, which is nationally recommended for SARS-CoV-2 detection in Japan (20). Viral RNA was extracted from 140-μl saliva specimens using a QIAsymphony RNA kit (Qiagen, Hilden, Germany) according to the manufacturer’s instructions. RT-qPCR amplification of the SARS-CoV-2 N protein gene was performed using the QuantiTect probe RT-PCR kit (Qiagen) with the following sets of primers and probes: the N-1 set, the forward primer 5′-CAC ATT GGC ACC CGC AAT C-3′, the reverse primer 5′-GAG GAA CGA GAA GAG GCT TG-3′, and the probe 5′-FAM-ACT TCC TCA AGG AAC AAC ATT GCC A-TAMRA-3′ (where FAM is 6-carboxyfluorescein and TAMRA is 6-carboxytetramethylrhodamine), and the N-2 set, the forward primer 5′-AAA TTT TGG GGA CCA GGA AC-3′, the reverse primer 5′-TGG CAG CTG TGT AGG TCA AC-3′, and the probe 5′-FAM-ATG TCG CGC ATT GGC ATG GA-TAMRA-3′ (20). A positive result with either or both of the primer and probe sets indicated the presence of viral RNA.

### Detection of viral RNA by direct RT-qPCR methods without RNA extraction.

Direct RT-qPCR methods without RNA extraction were performed using three commercial kits. Method A used a SARS-CoV-2 direct detection RT-qPCR kit (TaKaRa Bio Inc., Kusatsu, Japan), method B used an Ampdirect 2019 novel coronavirus detection kit (Shimadzu Corporation, Kyoto, Japan), and method C used a SARS-CoV-2 detection kit (Toyobo, Osaka, Japan) according to the manufacturers’ instructions. Kits for methods A and B were used with the primer sets recommended by the Centers for Disease Control and Prevention (CDC) (21). The kit for method C was used with the same primer sets as those used in the RT-qPCR LDT method. These methods are semiquantitative. A positive result, indicating the presence of SARS-CoV-2 RNA, was determined according to the cycle threshold (*C_T_*) value. *C_T_* values of <40 and 45 obtained using methods A and B, respectively, with the primer or probe set for SARS-CoV-2 indicated virus presence. For method C, *C_T_* values of <45 obtained with both the primer and the probe sets for SARS-CoV-2 alongside the internal control indicated a positive result.

The experimental details of the three direct RT-qPCR methods are presented below. For method A, each sample (8 μl) was mixed with sample preparation buffer (2 μl) and incubated for 5 min at room temperature and then at 95°C for 5 min. The reaction mix contained 35 μl of reaction buffer, including the primers and probe, and was added to 10 μl of a prepared sample. Thermal cycling included reverse transcription at 52°C for 5 min and 95°C for 10 s and then 40 cycles of denaturation at 95°C for 5 s and annealing/extension at 60°C for 30 s. For method B, each sample (5 μl) was mixed with sample preparation buffer (5 μl) and incubated at 90°C for 5 min. The reaction mix contained 15 μl of reaction buffer, including the primers and probe, and was added to 10 μl of a prepared sample. Thermal cycling included reverse transcription at 42°C for 10 min and 95°C for 1 min, 45 cycles of denaturation at 95°C for 5 s, and annealing/extension at 60°C for 30 s. For method C, each sample (3 μl) was mixed with sample preparation reagent (3 μl) and incubated at 95°C for 5 min. The reaction mix contained 40 μl reaction buffer, including the primers and probe, and was added to 6 μl of a prepared sample. Thermal cycling included reverse transcription at 42°C for 5 min, initial denaturation at 95°C for 10 s, 45 cycles of denaturation at 95°C for 5 s, and annealing/extension at 55°C for 10 s.

### Detection of viral RNA by an automated RT-qPCR device.

The cobas SARS-CoV-2 test (Roche, Basel, Switzerland) (22) was performed on the automated cobas 8800 RT-qPCR system (Roche) (3). Specimens (600 μl) were loaded onto the cobas 8800 with cobas SARS-CoV-2 master mix containing an internal RNA control, primers, and probes targeting the specific SARS-CoV-2 open reading frame 1 (ORF1) gene (target 1) and the envelope (E) gene (target 2). A cobas 8800 result positive for the presence of SARS-CoV-2 RNA was defined as “detected” if targets 1 and 2 were detected or “presumptively positive” if target 1 was not detected but target 2 was detected.

### Detection of viral RNA by RT-LAMP.

RT-LAMP detection of SARS-CoV-2 was performed using a Loopamp 2019-SARS-CoV-2 detection reagent kit (Eiken Chemical, Tokyo, Japan) according to the manufacturer’s instructions. The reaction was conducted at 62.5°C for 35 min with the turbidity-measuring real-time device LoopampEXIA (Eiken Chemical). A positive result for the LAMP reaction was determined automatically by LoopampEXIA based on turbidity.

### Detection of SARS-CoV-2 viral antigen by a rapid antigen test.

An RAT was performed using Espline SARS-CoV-2 (Fuji Rebio Inc.) according to the manufacturer’s instructions. In brief, the sample for analysis was obtained by dipping a swab, which was provided with the RAT kit, into the saliva specimen and then into the sample preparation mixture provided by the kit. The mixture (200 μl) was added to the sample port of the antigen assay. Subsequently, 2 drops of buffer were added, and the results were interpreted after a 30-min incubation.

### Definitions.

The saliva sample collection day was defined as day 1. Symptomatic cases were subdivided into two groups (23). Severe symptomatic cases were defined as patients showing clinical symptoms of pneumonia (dyspnea, tachypnea, a saturation of percutaneous oxygen [SpO_2_] level of <93%, and the need for oxygen therapy). Other symptomatic cases were classified as mild cases. The clinical signs and symptoms included all symptoms which occurred and were observed before saliva collection. A singleton test was conducted for each method.

### Ethical statement.

Written informed consent was obtained from each enrolled patient at the Self-Defense Forces Central Hospital. This study was reviewed and approved by the Self-Defense Forces Central Hospital (approval number 02-024) and the International University of Health and Welfare (20-Im-002-2).

### Statistical analysis.

Continuous variables with a normal distribution are expressed as means (± standard deviations [SD]) and, with a nonnormal distribution, as medians (interquartile ranges [IQR]) and were compared using Student's *t* test and the Wilcoxon rank sum test for parametric and nonparametric data, respectively. Categorical variables were expressed as numbers (percentages) and compared by the χ^2^ test or Fisher’s exact test. The Kruskal-Wallis test was used for nonparametric analysis with over three independent samples. Linear regression analysis was used to assess the relationship between each molecular diagnostic method. A two-sided *P* value of <0.05 was considered statistically significant. All statistical analyses were calculated using R (v3.4.0; R Foundation for Statistical Computing, Vienna, Austria [http://www.R-project.org/]).

## RESULTS

### Sensitivities of molecular diagnostic tests and the antigen test.

In this study, seven diagnostic tests for COVID-19 were compared across 103 saliva specimens, self-collected by 103 patients (Table 1, and see Table S1 in the supplemental material). Among the molecular diagnostic tests, the RT-qPCR LDT showed the highest sensitivity in analyzing the 103 saliva samples (81.6%), followed in order by the cobas SARS-CoV-2 test (80.6%), direct RT-qPCR method B (78.6%), method A (76.7%), RT-LAMP (70.9%), and method C (50.5%). Only 12 patients (11.7%) tested positive using the RAT.

**TABLE 1:** Summary of results of molecular diagnostic tests and the rapid antigen test for COVID-19 used on self-collected saliva samples

Test and primer set, method, or target	Total no. (%) of samples (95% confidence interval) (*n* = 103)	Total no. (%) of samples (95% confidence interval) at the indicated time of collection since the onset of symptoms		
		Early phase (≤9 days) (*n* = 61)	Late phase (>9 days) (*n* = 27)	No specific time (asymptomatic) (*n* = 15)
RT-qPCR LDT^*a*^	84 (81.6) (72.7–88.5)	57 (93.4) (84.1–98.2)	17 (63.0) (42.4–80.6)	10 (66.7) (38.4–88.2)
N-1 set^*e*^	76 (73.8) (64.2–82.0)	54 (88.5) (77.8–95.2)	14 (51.9) (31.9–71.3)	8 (53.3) (26.6–78.7)
N-2 set^*e*^	83 (80.6) (71.6–87.7)	57 (93.4) (84.1–98.2)	16 (59.3) (38.8–77.6)	10 (66.7) (38.4–88.2)
cobas SARS-CoV2 test	83 (80.6) (71.6–87.7)	56 (91.8) (81.9–97.3)	18 (66.7) (46.0–83.5)	9 (60.0) (32.3–83.7)
Target 1	76 (73.8) (64.2–82.0)	54 (88.5) (77.8–95.2)	14 (51.9) (31.9–71.3)	8 (53.3) (26.6–78.7)
Target 2	83 (80.6) (64.2–82.0)	56 (91.8) (81.9–97.3)	18 (66.7) (46.0–83.5)	9 (60.0) (32.3–83.7)
Direct RT-qPCR				
Method A^*b*^	79 (76.7) (67.3–84.5)	53 (86.9) (75.8–94.2)	16 (59.3) (38.8–77.6)	10 (66.7) (38.4–88.2)
Method B^*c*^	81 (78.6) (69.5–86.1)	55 (90.2) (79.8–96.3)	17 (63.0) (42.4–80.6)	9 (60.0) (32.3–83.7)
N-1 set^*f*^	80 (77.7) (68.4–85.3)	54 (88.5) (77.8–95.3)	17 (63.0) (42.4–80.6)	9 (60.0) (32.3–83.7)
N-2 set^*f*^	63 (61.2) (51.1–70.6)	48 (78.7) (66.3–88.1)	8 (29.6) (13.8–50.2)	7 (46.7) (21.3–73.4)
Method C^*d*^	52 (50.5) (40.5–60.5)	40 (65.6) (52.3–77.3)	6 (22.2) (8.6–42.3)	6 (40.0) (16.3–67.7)
N-1 set^*e*^	15 (14.6) (8.4–22.9)	9 (14.8) (7.0–26.1)	2 (7.4) (1.0–24.3)	4 (26.7) (7.8–55.1)
N-2 set^*e*^	51 (49.5) (39.5–59.5)	40 (65,6) (52.3–77.3)	6 (22.2) (8.6–42.3)	5 (33.3) (11.8–61.6)
RT-LAMP	73 (70.9) (61.1–79.4)	52 (85.2) (73.8–93.0)	12 (44.4) (25.5–64.7)	9 (60.0) (32.3–83.7)
Rapid antigen test	12 (11.7) (6.2–19.5)	8 (13.1) (5.8–24.2)	2 (7.4) (1.0–24.3)	2 (13.3) (1.7–40.5)

a LDT, laboratory-developed test.

b Method A, SARS-CoV-2 direct detection RT-qPCR kit (TaKaRa Bio Inc., Kusatsu, Japan).

c Method B, Ampdirect 2019 novel coronavirus detection kit (Shimadzu Corporation, Kyoto, Japan).

d Method C, SARS-CoV-2 detection kit (Toyobo, Osaka, Japan).

e Primer and probe set recommended by the National Institute of Infectious Diseases (NIID) in Japan.

f Primer and probe set recommended by the Centers for Disease Control and Prevention (CDC) in the United States.

### Correlation of molecular diagnostic tests and the antigen test.

**FIG 1:**
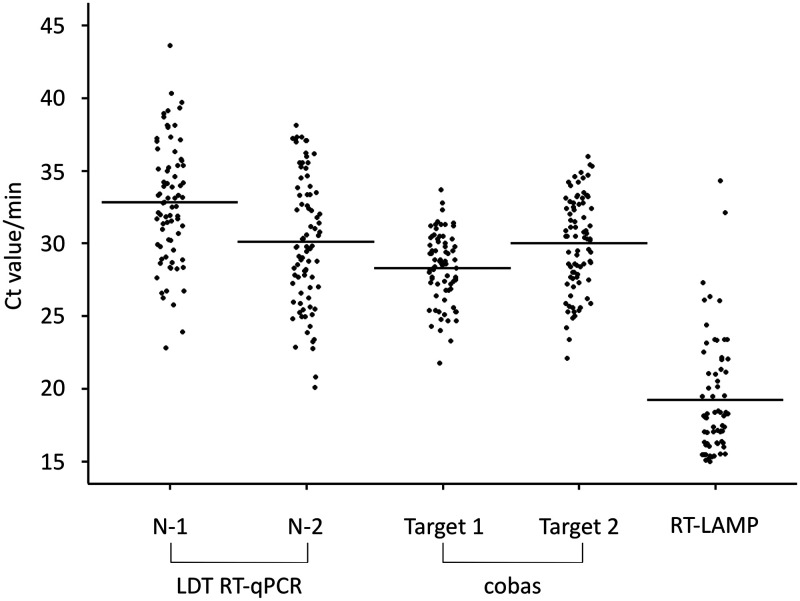
Cycle threshold (*C_T_*) values and detection times for each molecular diagnostic test of saliva specimens. *C_T_* value for each RT-qPCR primer set and detection time by reverse transcription–loop-mediated isothermal amplification (RT-LAMP). Horizontal lines indicate the mean *C_T_* value or detection time.

**FIG 2:**
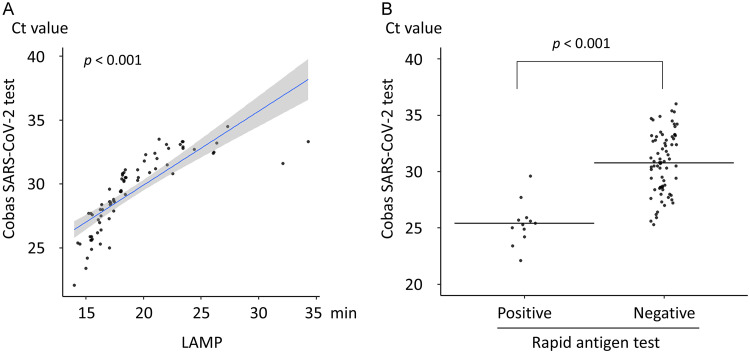
Relation of RT-qPCR, RT-LAMP, and the rapid antigen test (RAT) results for saliva specimens. (A) Relation between the detection time of reverse transcription–loop-mediated isothermal amplification (RT-LAMP) and the *C_T_* value of target 2 (SARS-CoV-2 envelope gene) in the cobas SARS-CoV-2 test. The blue slope line represents the fitted regression curve. The gray shadow indicates the 95% confidence interval around the regression curve. (B) Distribution of the *C_T_* values of target 2 for the cobas SARS-CoV-2 test of saliva with positive and negative results. Horizontal lines indicate the mean *C_T_* value. The *P* value was calculated using Student’s *t* test.

The mean *C_T_* values for the target 1 primer set (28.3 ± 2.3) for the cobas SARS-CoV-2 test were lower than those for N-1 (32.8 ± 4.1) and N-2 (30.1 ± 4.4) for the RT-qPCR LDT and target 2 (30.0 ± 3.2) for cobas SARS-COV-2 (Fig. 1). A significant correlation was observed between RT-LAMP detection time and the *C_T_* value of target 2 in the cobas SARS-CoV-2 test (*P < *0.001) (Fig. 2A). The *C_T_* value of target 2 in the cobas SARS-CoV-2 test was significantly lower in saliva samples that tested positive by the RAT than in samples that tested negative (25.4 ± 1.8 versus 30.8 ± 2.7, respectively; *P < *0.001) (Fig. 2B).

### Effect of collection time on test sensitivity.

On the day of admission, 15 patients (14.6%) who did not display any symptoms were classified as asymptomatic, whereas 88 patients (85.4%) were classified as COVID-19 symptomatic. Of the 88 symptomatic patients, saliva specimens were collected from 61 patients (69.3%) during the early phase of onset, defined as within 9 days of symptom onset (Table 1). Among these 61 early-phase patients, saliva samples were taken from 37 of them within 6 days of symptom onset (Table S1). Saliva samples were taken from the other 27 symptomatic patients (30.7%) after 10 days of symptoms, defined as the late phase of onset (Table 1). Samples from early-phase, late-phase, and asymptomatic patients tested positive by molecular diagnostic tests at percentages of 65.6 to 93.4%, 22.3 to 66.7%, and 40.0 to 66.7%, respectively. The detection of viral RNA in saliva was significantly higher in symptomatic patients who collected their saliva within 9 days of symptom onset than in saliva collected after 10 days of symptoms and in saliva from asymptomatic patients (*P < *0.01). There were no significant differences in prevalences of positive results by the RAT among the three groups. Among the original asymptomatic patients, four patients (26.7%) developed symptoms during the hospitalization period, and all four patients tested positive using each of the methods except for direct RT-qPCR method C and the RAT. The period from the day of saliva collection to symptom onset was 2 to 4 days.

### Effect of clinical background on the prevalence of viral RNA in saliva.

The baseline clinical characteristics of the 103 patients enrolled in this study are presented in Table 2. Briefly, patient age ranged from 18 to 87 years (median, 46 years; IQR, 38 to 63 years), and 66 (64.1%) patients were male. The time from symptom onset to sample collection was 1 to 14 days (median, 7 days; IQR, 6 to 10 days). The time from the initial RT-qPCR-positive test to sample collection was 1 to 8 days (median, 4 days; IQR, 3 to 5 days). Of the 88 symptomatic patients, 72 (81.8%) and 16 (18.2%) were classified as having mild and severe COVID-19, respectively.

**TABLE 2:** Effect of clinical background against the presence of viral RNA in the saliva of 103 asymptomatic and symptomatic patients

Parameter	Presence of viral RNA in saliva		
	Positive sample (*n* = 84)	Negative sample (*n* = 19)	*P* value^*a*^
Median age (yr) of subjects with indicated test result (IQR)	47 (39–67)	44 (38–55)	0.195

No. (%) of subjects with indicated test result/total who were:			
Male (*n* = 66)	58/66 (87.9)	8/66 (12.1)	0.035
Female (*n* = 37)	26/37 (70.3)	11/37 (29.7)	

No. (%) of subjects with indicated test result/total who were:			
Asymptomatic (*n* = 15)	10/15 (66.7)	5/15 (33.3)	0.146
Symptomatic (*n* = 88)	74/88 (84.1)	14/88 (15.9)	

a *P* values were calculated using the Wilcoxon rank sum test for continuous variables and the χ^2^ test or Fisher’s exact test for categorical variables.

The effect of clinical background against the prevalence of viral RNA in saliva was analyzed using the results of the RT-qPCR LDT, which had the highest sensitivity of all of the methods in this study. Among 103 patients, 66 (64.1%) were male and 37 (35.9%) were female. Of the 66 male patients, 58 (87.9%) tested positive by the RT-qPCR LDT, while 26 of 37 (70.3%) female patients tested negative (*P = *0.035) (Table 2). There were no significant differences in distribution by age or disease activity between patients in whose samples viral RNA was detected or undetected (*P > *0.05).

A summary of clinical symptoms and disease severity is shown for 88 symptomatic patients in Table 3. The disease symptom coughing was observed in 45 patients, and 41 of the 45 (91.1%) tested positive by the RT-qPCR LDT, while 4 of the 45 patients (8.9%) tested negative (*P = *0.084). All patients with severe disease (16/16, 100%) tested positive for viral RNA in their saliva, while 58 of 72 (78.4%) patients with mild disease tested positive (*P = *0.064).

**TABLE 3:** Effect of clinical background against the presence of viral RNA in saliva of 88 symptomatic patients

Parameter	Presence of viral RNA in saliva		*P* value^*a*^
	Positive (*n* = 74)	Negative (*n* = 14)	
Median age (yr) of subjects with indicated test result (IQR)	46 (38–60)	44 (37–53)	0.344

No. (%) of subjects with indicated test result/total who were:			
Male (*n* = 59)	54/59 (91.5)	5/59 (8.5)	0.011
Female (*n* = 29)	20/29 (69.0)	9/29 (31.0)	

No. (%) of subjects with indicated test result/total with disease severity:			
Mild (*n* = 72)	58/72 (80.6)	14/72 (19.4)	0.064
Severe (*n* = 16)	16/16 (100)		

No. (%) of subjects with indicated test result/total with clinical symptom:			
Fever (*n* =73)	62/73 (84.9)	11/73 (15.1)	0.700
Cough (*n* = 45)	41/45 (91.1)	4/45 (8.9)	0.084
Malaise (*n* = 30)	25/30 (83.3)	5/30 (16.7)	1.000
Headache (*n* = 25)	22/25 (88.0)	3/25 (12.0)	0.749
Diarrhea (*n* = 20)	15/20 (75.0)	5/20 (25.0)	0.294
Sore throat (*n* = 19)	15/19 (78.9)	4/19 (21.1)	0.491
Tachypnea (*n* = 13)	13/13 (100)		0.117
Dyspnea (*n* = 8)	8/8 (100)		0.346
Hypoxemia (SpO_2_ < 93%) (*n* = 10)	10/10 (100)		0.353

a The *P* value was calculated using the Wilcoxon rank sum test for continuous variables and the χ^2^ test or Fisher’s exact test for categorical variables.

## DISCUSSION

Here, we present evidence for the clinical usefulness of saliva specimens in diagnosing COVID-19. Previous studies reported that the sensitivities of RT-qPCR in analyzing saliva specimens for COVID-19 were 69.2 to 100%, compared with the initial diagnosis from throat and nasopharyngeal swabs initially collected from hospitalized patients (11–17, 19). The difference in sensitivity probably reflects differences in the clinical backgrounds of subjects and in timing of sampling in each study. In a comparison using RT-qPCR of nasopharyngeal swabs, Becker et al. reported the lowest sensitivity (69.2%) when clinical saliva samples that were collected in the late phase of onset were used (19). On the other hand, Azzi et al. reported the highest sensitivity with saliva samples (100%) among hospitalized patients with severe and very severe disease (12). In our study, the detection of viral RNA in saliva was significantly higher in samples collected in the early phase of symptom onset (within 9 days) than in samples collected in the late phase of symptom onset (over 10 days). Since the viral load of SARS-CoV-2 in saliva has been shown to decline from symptom onset (13), saliva specimens should be collected during the early phase of symptom onset to increase sensitivity.

The difference in saliva flow rate may affect the viral load in saliva and be associated with the difference in diagnostic sensitivity between males and females. We did not observe significant differences in disease severity or clinical symptoms between patients in whose saliva viral RNA was and was not detected; however, the prevalence of severe disease and the symptom of coughing were frequently observed in patients in whose saliva viral RNA was detected. Regarding disease activity, the presence of viral RNA was detected in more than 50% of the asymptomatic patients and the patients before the onset of symptoms. These findings support previous studies reporting the presence of viral RNA in the saliva of both symptomatic and asymptomatic patients (16). Therefore, our findings revealed that saliva, collected in the early phase of symptom onset, is a reliable and practical source for the screening and diagnosing of COVID-19.

The clinical performances of direct RT-qPCR kits and RT-LAMP and any correlation with RT-qPCR results were not well evaluated because of the small number of clinical specimens collected from patients in previous studies (4, 5, 24). The sensitivity of RT-LAMP for SARS-CoV-2 using upper and lower respiratory tract specimens has been reported as equivalent to that of RT-qPCR (4, 5, 24). However, our results indicate that the sensitivity of RT-LAMP is inferior to that of the RT-qPCR LDT and the cobas SARS-CoV-2 test for COVID-19 in saliva specimens. Direct RT-qPCR kits without an RNA extraction process can reduce the time, cost, and human resources needed to conduct the assay. However, we showed that there is a large difference in sensitivity among the direct RT-qPCR kits. It is necessary to pay attention to the false-negative results of RT-LAMP and direct RT-qPCR kits, especially when testing saliva samples. In clinical settings with limited medical and human resources, using RT-LAMP and direct RT-qPCR kits are options for screening and diagnosing COVID-19 because of their simplicity.

In comparison with molecular diagnostic tests, the SARS-CoV-2 RAT of saliva specimens showed low sensitivity. The sensitivity of the RAT is still unclear, not only when saliva samples but also when nasopharyngeal swab specimens are used (6). The experiment to compare the sensitivities of RT-qPCR and the RAT, prior to approval as an *in vitro* diagnostic test by the Japanese government, showed that the sensitivity of the RAT was 66.7% (16/24 patients) for nasopharyngeal swabs. Furthermore, low-sensitivity specimens contained a low viral copy number (50% sensitivity [6/12 patients] for specimens containing <100 copies/test) (6). Our findings also suggest that the RAT requires a high viral copy number to achieve positive results. The RAT kit was not originally compatible with saliva specimens and their viscosity; the freeze-thaw and centrifugation processes may have affected sensitivity. Improvements in sample preparation may increase its sensitivity.

Our study had several limitations. First, the saliva specimens were collected from patients 3 days (median) after receiving their first positive RT-qPCR result from an analysis of upper respiratory specimens. Directly comparing the sensitivities of tests using saliva and other upper or lower respiratory specimens is difficult in our study design because the viral loads in the clinical specimens vary with time (14). Second, although the high specificity of RT-qPCR and RT-LAMP for SARS-CoV-2 has been confirmed (5, 20, 24–27), the specificities should be analyzed by also using saliva from non-COVID-19 patients. Further studies are warranted to determine the usefulness of saliva specimens for screening and diagnosing COVID-19.

### Conclusions.

Self-collected saliva in the early phase of symptom onset is an alternative specimen option for diagnosing COVID-19. The RT-qPCR LDT, the cobas SARS-CoV-2 high-throughput system, direct RT-qPCR kits (except for one commercial kit), and RT-LAMP showed different sensitivities for detecting viral RNA in saliva specimens, but each can be selectively used according to the clinical setting and facilities if close attention is paid to any false-negative results. The rapid SARS-CoV-2 antigen test alone is not recommended for use at this time due to its low sensitivity.

## Supplementary Material

Supplemental file 1

## References

[1] Huang C, Wang Y, Li X, Ren L, Zhao J, Hu Y, Zhang L, Fan G, Xu J, Gu X, Cheng Z, Yu T, Xia J, Wei Y, Wu W, Xie X, Yin W, Li H, Liu M, Xiao Y, Gao H, Guo L, Xie J, Wang G, Jiang R, Gao Z, Jin Q, Wang J, Cao B 2020 Clinical features of patients infected with 2019 novel coronavirus in Wuhan, China. Lancet 395:497–506. doi:10.1016/S0140-6736(20)30183-5. 31986264 PMC7159299

[2] Sethuraman N, Jeremiah SS, Ryo A 2020 Interpreting diagnostic tests for SARS-CoV-2. JAMA 323:2249. doi:10.1001/jama.2020.8259. 32374370

[3] Cobb B, Simon CO, Stramer SL, Body B, Mitchell PS, Reisch N, Stevens W, Carmona S, Katz L, Will S, Liesenfeld O 2017 The cobas 6800/8800 system: a new era of automation in molecular diagnostics. Expert Rev Mol Diagn 17:167–180. doi:10.1080/14737159.2017.1275962. 28043179

[4] Yu L, Wu S, Hao X, Dong X, Mao L, Pelechano V, Chen WH, Yin X 2020 Rapid detection of COVID-19 coronavirus using a reverse transcriptional loop-mediated isothermal amplification (RT-LAMP) diagnostic platform. Clin Chem 66:975–977. doi:10.1093/clinchem/hvaa102. 32315390 PMC7188121

[5] Kitagawa Y, Orihara Y, Kawamura R, Imai K, Sakai J, Tarumoto N, Matsuoka M, Takeuchi S, Maesaki S, Maeda T 2020 Evaluation of rapid diagnosis of novel coronavirus disease (COVID-19) using loop-mediated isothermal amplification. J Clin Virol 129:104446. doi:10.1016/j.jcv.2020.104446. 32512376 PMC7241399

[6] Japanese Ministry of Health, Labour and Welfare. 2020 Approval of in vitro diagnostics for the novel coronavirus infection. Japanese Ministry of Health, Labour and Welfare, Tokyo, Japan https://www.mhlw.go.jp/content/11124500/000632304.pdf. Accessed 30 May 2020.

[7] Wehrhahn MC, Robson J, Brown S, Bursle E, Byrne S, New D, Chong S, Newcombe JP, Siversten T, Hadlow N 2020 Self-collection: an appropriate alternative during the SARS-CoV-2 pandemic. J Clin Virol 128:104417. doi:10.1016/j.jcv.2020.104417. 32403007 PMC7198188

[8] Wang W, Xu Y, Gao R, Lu R, Han K, Wu G, Tan W 2020 Detection of SARS-CoV-2 in different types of clinical specimens. JAMA 323:1843–1844. doi:10.1001/jama.2020.3786. 32159775 PMC7066521

[9] Yang Y, Yang M, Shen C, Wang F, Yuan J, Li J, Zhang M, Wang Z, Xing L, Wei J, Peng L, Wong G, Zheng H, Liao M, Feng K, Li J, Yang Q, Zhao J, Zhang Z, Liu L, Liu Y 2020 Evaluating the accuracy of different respiratory specimens in the laboratory diagnosis and monitoring the viral shedding of 2019-nCoV infections. medRxiv doi:10.1101/2020.02.11.20021493.

[10] Chan JF, Yip CC, To KK, Tang TH, Wong SC, Leung KH, Fung AY, Ng AC, Zou Z, Tsoi HW, Choi GK, Tam AR, Cheng VC, Chan KH, Tsang OT, Yuen KY 2020 Improved molecular diagnosis of COVID-19 by the novel, highly sensitive and specific COVID-19-RdRp/Hel real-time reverse transcription-PCR assay validated in vitro and with clinical specimens. J Clin Microbiol 58:e00310-20. doi:10.1128/JCM.00310-20. 32132196 PMC7180250

[11] Pasomsub E, Watcharananan SP, Boonyawat K, Janchompoo P, Wongtabtim G, Suksuwan W, Sungkanuparph S, Phuphuakrat A 15 5 2020 Saliva sample as a non-invasive specimen for the diagnosis of coronavirus disease-2019 (COVID-19): a cross-sectional study. Clin Microbiol Infect doi:10.1016/j.cmi.2020.05.001. PMC7227531 32422408

[12] Azzi L, Carcano G, Gianfagna F, Grossi P, Gasperina DD, Genoni A, Fasano M, Sessa F, Tettamanti L, Carinci F, Maurino V, Rossi A, Tagliabue A, Baj A 2020 Saliva is a reliable tool to detect SARS-CoV-2. J Infect 81:e45–e50. doi:10.1016/j.jinf.2020.04.005. 32298676 PMC7194805

[13] To KK-W, Tsang O-Y, Chik-Yan Yip C, Chan K-H, Wu T-C, Chan JMC, Leung W-S, Chik T-H, Choi C-C, Kandamby DH, Lung DC, Tam AR, Poon R-S, Fung A-F, Hung I-N, Cheng V-C, Chan J-W, Yuen K-Y 12 2 2020 Consistent detection of 2019 novel coronavirus in saliva. Clin Infect Dis doi:10.1093/cid/ciaa149. PMC7108139 32047895

[14] To KK-W, Tsang O-Y, Leung W-S, Tam AR, Wu T-C, Lung DC, Yip CC-Y, Cai J-P, Chan J-C, Chik T-H, Lau D-L, Choi C-C, Chen L-L, Chan W-M, Chan K-H, Ip JD, Ng A-K, Poon R-S, Luo C-T, Cheng V-C, Chan J-W, Hung I-N, Chen Z, Chen H, Yuen K-Y 2020 Temporal profiles of viral load in posterior oropharyngeal saliva samples and serum antibody responses during infection by SARS-CoV-2: an observational cohort study. Lancet Infect Dis 20:565–574. doi:10.1016/S1473-3099(20)30196-1. 32213337 PMC7158907

[15] McCormick-Baw C, Morgan K, Gaffney D, Cazares Y, Jaworski K, Byrd A, Molberg K, Cavuoti D 15 5 2020 Saliva as an alternate specimen source for detection of SARS-CoV-2 in symptomatic patients using Cepheid Xpert Xpress SARS-CoV-2. J Clin Microbiol doi:10.1128/jcm.01109-20. PMC7383538 32414838

[16] Kojima N, Turner F, Slepnev V, Bacelar A, Deming L, Kodeboyina S, Klausner JD 2020 Self-collected oral fluid and nasal swabs demonstrate comparable sensitivity to clinician collected nasopharyngeal swabs for Covid-19 detection. medRxiv doi:10.1101/2020.04.11.20062372. PMC7665422 33075138

[17] Wyllie AL, Fournier J, Casanovas-Massana A, Campbell M, Tokuyama M, Vijayakumar P, Geng B, Muenker MC, Moore AJ, Vogels CBF, Petrone ME, Ott IM, Lu P, Lu-Culligan A, Klein J, Venkataraman A, Earnest R, Simonov M, Datta R, Handoko R, Naushad N, Sewanan LR, Valdez J, White EB, Lapidus S, Kalinich CC, Jiang X, Kim DJ, Kudo E, Linehan M, Mao T, Moriyama M, Oh JE, Park A, Silva J, Song E, Takahashi T, Taura M, Weizman O-E, Wong P, Yang Y, Bermejo S, Odio C, Omer SB, Dela Cruz CS, Farhadian S, Martinello RA, Iwasaki A, Grubaugh ND, Ko AI 2020 Saliva is more sensitive for SARS-CoV-2 detection in COVID-19 patients than nasopharyngeal swabs. medRxiv doi:10.1101/2020.04.16.20067835.

[18] To KK-W, Yip CC-Y, Lai C-W, Wong C-H, Ho D-Y, Pang P-P, Ng A-K, Leung KH, Poon R-S, Chan KH, Cheng V-C, Hung I-N, Yuen KY 2019 Saliva as a diagnostic specimen for testing respiratory virus by a point-of-care molecular assay: a diagnostic validity study. Clin Microbiol Infect 25:372–378. doi:10.1016/j.cmi.2018.06.009. 29906597

[19] Becker D, Sandoval E, Amin A, De Hoff P, Diets A, Leonetti N, Lim YW, Elliott C, Laurent L, Grzymski J, Lu J 2020 Saliva is less sensitive than nasopharyngeal swabs for COVID-19 detection in the community setting. medRxiv doi:10.1101/2020.05.11.20092338.

[20] Shirato K, Nao N, Katano H, Takayama I, Saito S, Kato F, Katoh H, Sakata M, Nakatsu Y, Mori Y, Kageyama T, Matsuyama S, Takeda M 18 2 2020 Development of genetic diagnostic methods for novel coronavirus 2019 (nCoV-2019) in Japan. Jpn J Infect Dis doi:10.7883/yoken.JJID.2020.061. 32074516

[21] Centers for Disease Control and Prevention. 2020 Research use only 2019-novel coronavirus (2019-nCoV) real-time RT-PCR primers and probes. Centers for Disease Control and Prevention, Atlanta, GA https://www.cdc.gov/coronavirus/2019-ncov/lab/rt-pcr-panel-primer-probes.html. Accessed 30 May 2020.

[22] US Food and Drug Administration. 2020 cobas® SARS-CoV-2. US Food and Drug Administration, Silver Spring, MD https://www.fda.gov/media/136049/download. Accessed 30 May 2020.

[23] Zhang J, Zhou L, Yang Y, Peng W, Wang W, Chen X 2020 Therapeutic and triage strategies for 2019 novel coronavirus disease in fever clinics. Lancet Respir Med 8:e11–e12. doi:10.1016/S2213-2600(20)30071-0. 32061335 PMC7159020

[24] Yan C, Cui J, Huang L, Du B, Chen L, Xue G, Li S, Zhang W, Zhao L, Sun Y, Yao H, Li N, Zhao H, Feng Y, Liu S, Zhang Q, Liu D, Yuan J 2020 Rapid and visual detection of 2019 novel coronavirus (SARS-CoV-2) by a reverse transcription loop-mediated isothermal amplification assay. Clin Microbiol Infect 26:773–779. doi:10.1016/j.cmi.2020.04.001. 32276116 PMC7144850

[25] Lieberman JA, Pepper G, Naccache SN, Huang M-L, Jerome KR, Greninger AL 29 4 2020 Comparison of commercially available and laboratory developed assays for in vitro detection of SARS-CoV-2 in clinical laboratories. J Clin Microbiol doi:10.1128/JCM.00821-20. PMC7383518 32350048

[26] Pfefferle S, Reucher S, Nörz D, Lütgehetmann M 2020 Evaluation of a quantitative RT-PCR assay for the detection of the emerging coronavirus SARS-CoV-2 using a high throughput system. Euro Surveill 25:2000152. doi:10.2807/1560-7917.ES.2020.25.9.2000152. PMC7068162 32156329

[27] Okamaoto K, Shirato K, Nao N, Saito S, Kageyama T, Hasegawa H, Suzuki T, Matsuyama S, Takeda M 30 4 2020 An assessment of real-time RT-PCR kits for SARS-CoV-2 detection. Jpn J Infect Dis doi:10.7883/yoken.JJID.2020.108. 32350226

